# Acute Hemoperitoneum With Hemorrhagic Shock in Late Pregnancy: Spontaneous Splenic Artery Rupture Presenting as a Non‐Obstetric Abdominal Emergency

**DOI:** 10.1002/ccr3.73168

**Published:** 2026-07-18

**Authors:** Annalisa Di Cello, Manfredo Tedesco, Francesca Diaco, Anna Monardo, Agostino Siciliano, Carmelina Donatella Ermio

**Affiliations:** ^1^ Unit of Obstetrics and Gynecology, Maternal and Child Health Department Lamezia Terme Hospital, ASP of Catanzaro Lamezia Terme Italy; ^2^ Department of Surgery Giovanni Paolo II Hospital, ASP Catanzaro Lamezia Terme Italy; ^3^ Unit of Obstetrics and Gynecology, Department of Clinical and Experimental Medicine Magna Graecia University Catanzaro Italy; ^4^ Department of Anesthesia and Intensive Care Unit Lamezia Terme Hospital, ASP Catanzaro Lamezia Terme Italy; ^5^ Radiology Unit Dulbecco University Hospital Catanzaro Italy

**Keywords:** hemoperitoneum, hemorrhagic shock, maternal near miss, pregnancy, splenic artery rupture

## Abstract

Spontaneous splenic artery rupture is a rare but life‐threatening cause of hemoperitoneum in pregnancy. Rapid recognition and multidisciplinary management are essential. In hemodynamically unstable patients, emergency surgical exploration and individualized decisions regarding delivery may be necessary to optimize maternal stabilization and improve maternal and fetal outcomes.

## 1 Introduction

Hemoperitoneum during pregnancy is most commonly related to obstetric conditions such as placental abruption or uterine rupture. However, rare non‐obstetric etiologies may also result in catastrophic intra‐abdominal hemorrhage. Among these, splenic artery rupture represents an exceptionally uncommon but frequently fatal event [1, 2].

Splenic artery rupture in pregnancy is most often associated with aneurysmal degeneration. Historical series report maternal mortality exceeding 50% and fetal mortality approaching 70%–90% [1, 2, 3]. Although improvements in surgical and critical care management have reduced mortality in contemporary reports, outcomes remain highly dependent on rapid recognition and multidisciplinary intervention [4, 5, 6, 7].

**FIGURE 1:**
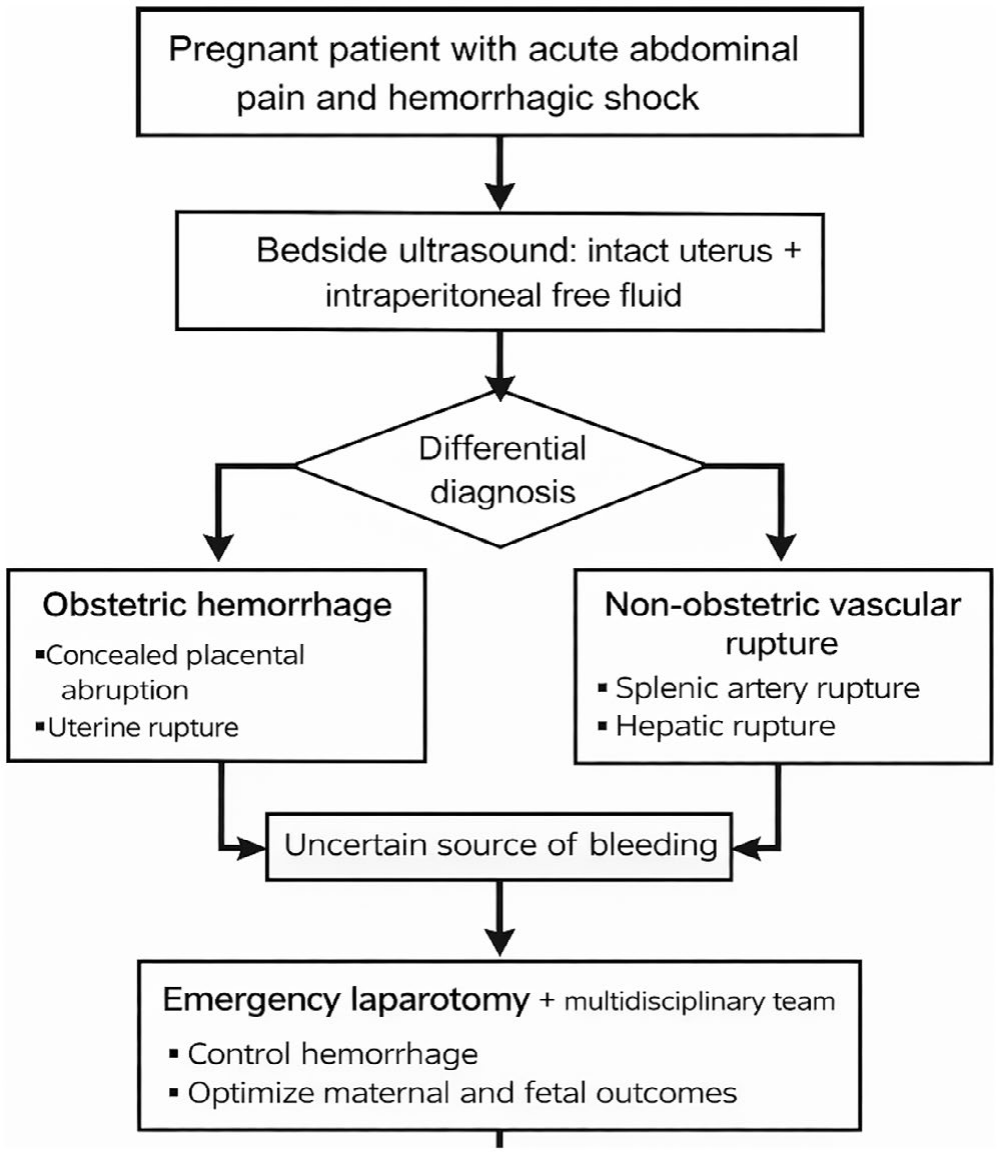
Diagnostic pathway for hemoperitoneum with hemorrhagic shock in pregnancy. Schematic representation of the clinical decision‐making process in pregnant patients presenting with acute abdominal pain and hemodynamic instability. The presence of free intraperitoneal fluid with an intact uterus should prompt consideration of non‐obstetric causes of hemoperitoneum and may require emergency surgical exploration.

In contrast to aneurysmal rupture, atraumatic non‐aneurysmal splenic rupture has also been described, although its precise pathophysiological mechanisms remain incompletely understood [8]. Pregnancy‐related increases in circulating blood volume and cardiac output may contribute to vessel wall stress, but causality cannot be definitively established [1, 8]. Proposed mechanisms potentially contributing to splenic artery rupture during pregnancy are illustrated in Figure 1.

The World Health Organization defines maternal near miss as a woman who nearly died but survived a life‐threatening complication during pregnancy [9]. Splenic artery rupture during pregnancy exemplifies such a scenario, particularly when presentation mimics more common obstetric emergencies.

## 2 Case History/Examination

A 26‐year‐old gravida 3 para 2 woman at 27 + 2 weeks' gestation presented with sudden‐onset epigastric pain occurring at rest shortly after food intake, without preceding trauma or exertion. The pain was followed by collapse at home. On admission she was hypotensive (80/50 mmHg), tachycardic (120 beats/min), pale, and diaphoretic. Hemoglobin was 6.8 g/dL, lactate 4.2 mmol/L, and metabolic acidosis was present. Findings were consistent with class III–IV hemorrhagic shock. Bedside ultrasound performed in the obstetric emergency unit confirmed fetal viability and demonstrated moderate‐to‐large intraperitoneal free fluid. The placenta appeared normally located without sonographic evidence of placental abruption. No uterine wall discontinuity or sonographic findings suggestive of uterine rupture were identified. Fetal heart rate was within the normal range at the initial assessment. There was no vaginal bleeding. The pregnancy had been uncomplicated, without hypertensive disorders, preeclampsia, fetal growth restriction, or other significant obstetric complications.

## 3 Differential Diagnosis, Investigations and Treatment

Differential diagnosis included concealed placental abruption, uterine rupture, hepatic rupture, trauma‐related hemorrhage [4, 10], and visceral artery rupture. Although concealed placental abruption was initially considered because of maternal shock, the absence of vaginal bleeding, normal placental appearance on ultrasound, and the presence of diffuse intraperitoneal free fluid suggested an alternative source of hemorrhage.

Given progressive hypotension despite fluid resuscitation, emergency laparotomy was undertaken. A Pfannenstiel incision was selected based on the initial suspicion of obstetric hemorrhage. Once a non‐obstetric source of hemorrhage was identified, the procedure was extended to allow adequate upper abdominal exploration in collaboration with the general surgery team. Approximately 3–4 L of hemoperitoneum was encountered, with an intact uterus.

Upon identification of massive hemoperitoneum with an intact uterus, a multidisciplinary intraoperative response was immediately initiated. While maternal resuscitation was ongoing, the general surgery team was urgently activated. Given persistent hemodynamic instability and the need to optimize surgical exposure, cesarean delivery was performed to facilitate maternal stabilization and operative access. At the time of surgery, the profound maternal shock raised concerns regarding ongoing fetal compromise secondary to severely reduced uteroplacental perfusion. Delivery also provided improved exposure of the upper abdomen, facilitating rapid identification and control of the bleeding source.

The sequence of interventions was guided by dynamic intraoperative reassessment of maternal hemodynamic status and surgical priorities, rather than by a fixed protocol. Massive transfusion protocol was activated. The patient received 5 units of packed red blood cells, 2 units of fresh frozen plasma, and 2 g fibrinogen.

As upper abdominal bleeding became evident: active arterial bleeding from the splenic artery was identified and immediately controlled by splenectomy with arterial ligation by the general surgeon who joined the operative field.

Histopathology showed no aneurysmal degeneration or vasculitis. Postoperative CT angiography excluded additional visceral aneurysms.

## 4 Conclusion and Results (Outcome and Follow‐Up)

Emergency laparotomy was initiated within 20 min of admission. Importantly, within 30 min of hospital admission, the source of hemorrhage had been identified and definitive surgical treatment with splenectomy and splenic artery ligation was underway, highlighting the time‐critical nature of this condition. The patient stabilized in the intensive care unit within 24 h. Preventive angiographic embolization was performed following vascular surgery recommendation to exclude residual arterial flow or occult vascular abnormalities. The neonate required NICU admission for prematurity‐related care and was discharged after six weeks without major complications.

## 5 Discussion

Spontaneous splenic artery rupture during pregnancy represents a rare but life‐threatening cause of non‐obstetric hemoperitoneum. Although most cases are associated with aneurysmal degeneration, non‐aneurysmal rupture has also been reported and may occur in the absence of identifiable structural pathology [1, 8]. In the present case, histopathology excluded aneurysmal changes and vasculitis, suggesting a spontaneous vascular event.

Several cases of splenic artery rupture during pregnancy have been reported in the literature. Ornaghi et al. described four obstetric cases with variable presentation and outcomes, highlighting the importance of rapid multidisciplinary management [11]. Similarly, Jacobson et al. reported splenic artery aneurysm rupture presenting with sudden abdominal pain and hemodynamic collapse during pregnancy [12]. Although most published cases involve aneurysmal rupture, non‐aneurysmal spontaneous rupture has also been described, emphasizing the diagnostic difficulty when no preexisting vascular lesion is identified [8]. Our case differs in that histopathology did not demonstrate aneurysmal degeneration, suggesting a spontaneous vascular event in the absence of underlying structural pathology.

Historically, maternal mortality in splenic artery rupture during pregnancy has exceeded 50%, with fetal mortality approaching 70%–90% [1, 2, 3]. Reported maternal and fetal mortality from selected series is summarized in Table 1.

**TABLE 1:** Selected reported outcomes of splenic artery rupture in pregnancy.

Study	Year	Study type	Maternal mortality	Fetal mortality
Sadat et al.	2008	Systematic review	25%–36%	50%–75%
Abbas et al.	2002	Institutional series	≈20%–30%	Not specified
Ginsburg et al.	1987	Literature review	> 50%	70%–90%
Ornaghi et al.	2022	Case series	Improved survival	Variable

*Note:* Data derived from published case series and systematic reviews. Mortality varies according to gestational age, timing of diagnosis, and availability of multidisciplinary care.

Contemporary reports suggest improved survival, likely reflecting advances in surgical, anesthetic, and critical care management [6, 7, 11, 12, 13, 14]. Nevertheless, outcomes remain highly dependent on rapid operative intervention.

Diagnosis is challenging because presentation may mimic more common obstetric emergencies. Acute abdominal pain with hemoperitoneum in pregnancy requires consideration of placental abruption, uterine rupture, hepatic rupture, trauma‐related hemorrhage, and visceral artery rupture [4, 10]. In our patient, concealed placental abruption and uterine rupture represented the leading initial diagnostic hypotheses because they are the most common causes of maternal shock associated with abdominal pain in late pregnancy. However, the absence of definitive obstetric findings together with progressive hemodynamic collapse prompted immediate exploratory laparotomy, which ultimately revealed a non‐obstetric source of hemorrhage.

One of the most debated aspects in splenic artery rupture during pregnancy concerns the timing of delivery. Some authors recommend delaying delivery until vascular control has been achieved to avoid adding a second potential bleeding source [5]. However, clinical scenarios vary significantly.

In our patient, persistent maternal hemodynamic instability despite fluid resuscitation and the need for improved surgical exposure influenced the decision to proceed with cesarean delivery. The choice was individualized, based on real‐time intraoperative assessment rather than adherence to a fixed surgical sequence.

Delivery in this context should not be interpreted as universally mandatory in all cases of hemoperitoneum with shock. Rather, it may be considered when maternal instability compromises uteroplacental perfusion, when surgical access is hindered by the gravid uterus, or when rapid obstetric intervention facilitates coordinated multidisciplinary management.

The favorable outcome in this case likely reflects prompt activation of a multidisciplinary team involving obstetrics, anesthesiology, general surgery, and interventional radiology. Early anesthesiologic management, including aggressive resuscitation and hemodynamic stabilization, was crucial to allow definitive surgical control of hemorrhage.

This case fulfills the World Health Organization criteria for maternal near miss [9], highlighting the importance of structured team response in rare but catastrophic vascular emergencies during pregnancy.

## Author Contributions

**Annalisa Di Cello:** conceptualization, investigation, methodology, validation, formal analysis, data curation, writing – original draft, writing – review and editing. **Agostino Siciliano:** methodology, validation, data curation, investigation. **Anna Monardo:** methodology, supervision, data curation. **Manfredo Tedesco:** methodology, data curation, writing – original draft, resources. **Carmelina Donatella Ermio:** investigation, conceptualization, writing – original draft, supervision, data curation, resources. **Francesca Diaco:** conceptualization, investigation, writing – original draft, formal analysis, writing – review and editing.

## Funding

The authors have nothing to report.

## Consent

Written informed consent was obtained from the patient for publication of this case report.

## Conflicts of Interest

The authors declare no conflicts of interest.

## Data Availability

The data that support the findings of this study are available on request from the corresponding author. The data are not publicly available due to privacy or ethical restrictions.
